# Adjunct Methods of the Standard Diabetic Foot Ulceration Therapy

**DOI:** 10.1155/2013/243568

**Published:** 2013-06-13

**Authors:** Dariusz Waniczek, Andrzej Kozowicz, Małgorzata Muc-Wierzgoń, Teresa Kokot, Elżbieta Świętochowska, Ewa Nowakowska-Zajdel

**Affiliations:** ^1^Department of General and Gastrointestinal Surgery, Silesian Medical University, 41-902 Bytom Katowice, Poland; ^2^Department of Internal Medicine, Silesian Medical University, 41-902 Bytom Katowice, Poland; ^3^Department of Biochemistry, Silesian Medical University, 41-800 Zabrze Katowice, Poland

## Abstract

The outcome of management of diabetic foot ulceration (DFU) is poor and insufficient. DFU therapy includes the standard management as debridement of the wound, revascularization procedures, off-loading of the ulcer and antibacterial actions, and supplementation of growth factors and cytokines, leading to stimulation of granulation, epidermization, and angiogenesis. The aim of the present review is to summarize the adjunct methods of the standard DFU therapy as hyperbaric oxygen therapy (HBOT), maggot therapy (MT), and platelet-rich plasma therapy (PRPT). The results of preclinical and clinical trials indicated that the methods may reduce time of therapy, short-term morbidity, and the risk of major amputation.

## 1. Introduction

Diabetes mellitus (DM) is one of the most deceitful diseases that affect more than 371 million people all over the world in 2012; by 2030 this will rise to 552 million [1]. The disease often leads to the development of serious, health threatening complications [2]. Of all diabetic complications, diabetic foot syndrome (DFS) is one of the most devastating and costly [2, 3]. According to the WHO, DFS are an infection, ulceration, and/or destruction of deep tissue, that comes along with neurologic abnormalities and/or different stages of arterial closure disease in the lower limbs. The International Consensus on the Diabetic Foot defined a diabetic foot ulcer as a full thickness wound below the ankle in a person with diabetes, irrespective of duration [4].

Diabetic foot ulceration (DFU) develops in 15–25% of DM patients. Approximately 15–25% of those cases require amputation [4, 5]. Some estimates have stated that the likelihood of amputation is 25–30 times higher among patients with diabetes than in the general population. Various studies have shown that the rates of major amputation of the diabetic foot are now decreasing at the regional level with rates declining from around 550 to 160–360 per 100,000 patients with diabetes, but the rates of minor amputation (toe/forefoot) have not changed [6].

 Ethiopathogenetic factors of DFU involve neuropathic, ischaemic, mechanic, metabolic and systemic risk factors, and infection (superinfection). Peripheral neuropathy is the most important causal pathway leading to foot ulceration and often leads to sensory deficit with the loss of protective pain sensation. Ischemia, on the other hand, results from atherosclerotic peripheral vascular disease, which usually affects the distal vessels of the lower limb [7, 8]. Infection can complicate any type of diabetic foot ulcer and is one of the most common causes of hospital admission among people with diabetes. Pathogenetic factors include the increased collagen deposition and network by advanced glycosylation end products, the loss of adipose tissue, and the occurrence of edema, which destroy the compensating balance between preventive and damaging factors.

 Neuropathic changes occur in approximately 85% [6] and ischaemic in 10–60% of cases [9, 10]. DFU is often associated with a secondary bacterial infection causing inflammation of the skin, subcutaneous tissue, muscles, tendons, and bones leading to necrosis of those tissues. Chronic ulceration or limb amputation, besides mutilation of a patient, is associated also with reduction of his/her dexterity and quality of life and other numerous threats for the patient's life and health [11, 12].

 Therapy of DFU complications constitutes also a social and economic problem [13]. Treatment requires the know-how of a specialized center in collaboration with different medical disciplines, for example, a diabetologist, surgeon, vascular surgeon, orthopaedist, radiologist, educator, shoemaker, and kinesiotherapist [4, 14]. The main purpose of that multidisciplinary foot care team is the prevention of DFU and its prompt therapy if the condition develops. The basic principles of prevention and treatment described in these guidelines are based on the International Consensus of the Diabetic Foot [4]. The standard of care for treating DFU includes optimization of glycemic control levels, appropriate nutrition, extensive debridement, infection elimination and dressings, and pressure relief in the areas of the foot. DFS therapy, following a possible vascular reconstruction, may consider a preventive surgery aimed at reduction of ulceration risk [14, 15]. However, even the best preventive management cannot exclude DM complications, and even the best DFU management based on standardised procedures gives no guarantee of a cure [10–15].

Knowledge regarding wound healing in DM patients has been rapidly expanding lately. It is a result of the development of new research on advanced therapeutic products, including stem cells, growth factors, skin substitutes, and gene therapy. Despite some promising results their efficacy remains unsatisfactory, and their combinations with the standard therapy often fails.

Interest is aroused by relatively efficient adjunct DFU treatment methods, including hyperbaric oxygen therapy (HBOT), maggot therapy (MT), and platelet-rich plasma therapy (PRPT). Those methods have been developing rapidly since the 1980s. The main reason for interest in those methods was the observation of rapidly increasing bacterial antibiotic resistance consequently leading to resignation of topical antibiotic application.

Wound healing is a complex and dynamic process in which the following factors play significant roles:

1. inflammation and associated immunological processes,
2. granulation and epidermization and associated cytokines and growth factors,
3. bioregulator-stimulated neoangiogenesis process.

Therefore, the main aims of DFU therapy include antibacterial actions and supplementation of growth factors and cytokines, leading to stimulation of granulation, epidermization, and angiogenesis [16].

## 2. Hyperbaric Oxygen Therapy (HBOT)

For over 50 years, HBOT has been a method applied to selected, serious cases of nonhealing, and infection-complicated DFU resistant to other therapeutic methods. Application of the method was initially based on theoretical assumptions and then on experimental research [17–20].

HBOT comprises patient inhalation with pure oxygen at the pressure of 2-3 absolute atmospheres ATAs (1 ATA = 14.7 psi, 1 kg per square centimeter, 101.3 kPa, 760 torr, or 760 mm Hg) provided by appropriate single- and multipatient pressure chambers. A single session lasts for 70–120 minutes, usually 90 minutes, and the number of sessions usually exceeds 20. HBOT-related complications are rare and involve claustrophobia, ear, sinus, or lung damage due to the pressure, temporary worsening of short sightedness, and oxygen poisoning [21]. Besides the commonly known relative and absolute contraindications, transcutaneous oximetry (TcPO2) is considered an additional criterion of classification for HBOT, treated as a valuable prognostic factor for ulceration treated with the method [22]. In DFU patients, the TcPO2 method-measured oxygen pressure over 400 mm Hg at 2.5 ATA or over 50 mm Hg in pure oxygen environment at normal atmospheric pressure should be perceived as a good prognostic index [23–25].

Precise mechanism of action of HBOT in DFU healing has not been uncovered yet. Increased oxygen levels in wound environment instigate healing by a mechanism of angiogenesis. The process involves physical dissolution of oxygen in plasma, leading to increased supply of oxygen to hypoxia-affected tissues. In DFU pathogenesis, local and systemic metabolic disorders lead to abnormal oxygen supply to affected tissues, affecting locally the immunological system and favouring wound infection. Reduced activity of phagocytic macrophages, reduced chemotaxis, and adhesion of neutrophils are observed in DFU. Reduced immunity of tissues favours development of pathogenic bacterial flora, including anaerobic microorganisms. They release toxins causing hypoxia and oedema of tissues [26–29].

Hyperbaric chamber has a bactericidal and bacteriostatic effect. Oxygen administered under increased ambient pressure enhances *in vitro* phagocytosis in regions of limited perfusion by increasing local oxygen tension to levels consistent with normal phagocytic function [21]. At the pressure of 2.5 ATA and respiration with 100% oxygen, its tension in the plasma may be as high as 2000 mm Hg, causing a 10–15-fold increase in oxygen transport, a 4-fold increase in oxygen diffusion to tissues on the arterial side, and a double increase on the venous side of the capillary circulation [20, 21].

Oxygen is an important cellular signal regulating intracellular and intratissue transformations. Increased oxygen level in chronically hypoxic or ischaemic wounds stimulates proliferation and differentiation of epithelial cells and fibroblasts and collagen synthesis in fibroblasts. Oxygen is a potent proangiogene. The element increases neovascularisation by angiogenic stimulation leading to new blood vessel formation from local endothelial cells and by the stimulation of the systemic stem/progenitor cells to differentiate in the form of blood vessels [18, 20–23, 27, 28]. It was demonstrated that HBOT stimulates vasculogenic stem cell mobilisation from bone marrow and recruits them to skin wound [29]. Increased tissue oxygenation during HBOT improves also tolerance to ischemia and reduces metabolic abnormalities in those tissues [30, 31].

The first broadly commented results of a nonrandomised clinical trial were published in 1987 by Baroni et al. [17]. The authors reported that 89% of patients with DFU endangered by amputation because of necrotic changes (16 of 18 patients) healed in the HBO group, whereas only 60% (6 of 10 patients) healed in the control group (only treated with standard therapy). The control group included patients who did not consent to additional application of HBOT (Table 1).

Case reports, case series, case control studies, and randomised controlled trials presented in the end of the 20th century encouraged the application of HBOT [37–41] (Table 1). However, those studies were accused of methodology differences, including, among others, lack of inclusion and exclusion criteria.

From the methodological point of view, the strongest evidence of HBOT efficacy is offered by the randomized, double-blinded, and placebo-controlled clinical trial by Abidia et al. [35] (Table 1), but the study was small and included only patients with Wagner grade 1 and 2 ulcers [15] (Table 2). The authors demonstrated a significant decrease of the wound areas in the treatment group in comparison to the control group. Moreover, the cost-effectiveness analysis has shown a potential saving in the total cost of treatment with HBOT for each patient during the study.

The study by Kalani et al. included 38 patients with ischemic ulcers without full-thickness gangrene. After three years, 76% of the 17 patients receiving HBOT had healed their ulcers to intact skin compared with 48% of those given conventional treatment [36] (Table 1). In the randomized trial by Kessler et al. [68], the effect of two daily 90 min sessions of HBOT five days a week for two weeks was compared with regular treatment in 28 hospitalized patients with neuropathic Wagner grade 1 to 3 ulcers. After two weeks of treatment, the reduction in ulcer area was doubled in the HBOT group. However, this improvement disappeared during the next two weeks of followup.

In the unblinded, randomized study by Duzgun et al. [34], the effect of HBOT was compared with standard therapy in 100 patients with a foot ulcer duration of at least four weeks (Table 1). During a mean follow-up period of 92 weeks, primary healing was achieved in 66% of patients receiving HBOT compared with 0% following standard therapy. A review of 6 studies prepared by Roeckl-Wiedmann et al. [69] demonstrated that additional application of HBOT reduced the risk of amputation in 118 patients. Other double-blinded, randomized, and placebo-controlled clinical trials presented by Löndahl et al. [33, 70, 71] proved applicability of HBOT in DFU adjunct therapy. Complete healing of the index ulcer (acc. Wagner scales) was achieved in 37 patients at 1 year of followup in 25/48 (52%) in the HBOT group and 12/42 (29%) in the placebo group (Table 1).

The Cochrane database systemic review, based on 8 studies of DFU (455 participants), demonstrated a favourable effect of HBOT on wound healing on the early stage of healing (6 weeks), but a longer observation (one year) failed to demonstrate any long-standing positive effects of the therapy. Additional application of HBOT had no significant effect on reduction of the number of major amputations [32].

Liu et al. [72] summarize thirteen trials (a total of 624 patients), including 7 prospective randomized trials, performed between January 1, 1966, and April 20, 2012. Pooling analysis revealed that, compared with treatment without HBO, adjunctive treatment with HBO resulted in a significantly higher proportion of healed diabetic ulcers (relative risk, 2.33; 95% CI, 1.51–3.60). The analysis also revealed that treatment with HBO was associated with a significant reduction in the risk of major amputations (relative risk, 0.29; 95% CI, 0.19–0.44); however, the rate of minor amputations was not affected. Adverse events associated with HBO treatment were rare and reversible and not more frequent than those occurring without HBO treatment.

 DFU infections are often asymptomatic and for that reason systemic antibiotic therapy is used earlier in their case than it is for other wounds. That long-standing therapy favours bacterial antibiotic resistance. Early introduction of HBOT reduces the risk of infection and the risk of amputation [73].

HBOT does not substitute the antibiotic therapy, local humid therapy, or surgical wound debridement. It may only support the complex DFU therapy. Maybe soon HBOT will become a routine procedure in standard DFU therapy, but still standardised procedures and therapy cost estimates are lacking. Some authors believe that HBOT should be introduced to standard therapy as early as possible, without months of delay spent on ineffective therapy.

## 3. Maggot Therapy (MT)

Successful DFU therapy largely depends on regular wound debridement and creation of favourable humid conditions free from bacterial infection. Physical-mechanic means are the simplest ones to be used for wound debridement. During a surgical procedure, necrotic tissue, fibrin, and pathological granulation are removed with a scalpel, scissors, and a scraper (surgical debridement). However, the method is associated with a risk of intense bleeding; it is painful and imprecise. Debridement often extends beyond the necessary boundary, as it is difficult to separate and differentiate necrotic tissue, granulation, or poorly perfused tissue from a healthy one. That is particularly important in case of DFU, where debridement is a common procedure and the wound's area is small [74]. Therefore, other, superior and more efficient methods of local therapy are sought. Among them, there are alternative physical methods of wound debridement: sonotherapy (with use of ultrasounds) and hydrosurgical (with use of water jets). They seem less efficient than MT—a method known for centuries.

 Therapeutic effect of MT is based in three mechanisms:

1. removal of necrotic tissue from the wound,
2. antibacterial effect and destruction of bacterial biofilm,
3. stimulation of healing processes.

Maggots secrete digestive juices on the outside; necrotic tissue becomes digested and liquefied and absorbed in that form. Additionally, an additional mechanical debridement is caused by the specific mandibles or “mouth hooks” of the maggots and their rough body which both scratch the necrotic tissue. Studies are on the way on chemical composition and mechanism of action of maggot excretions and secretions (ES). It is a blend of collagenases, proteolytic enzymes, serine proteases: trypsin-like and chymotrypsin-like enzymes, metalloproteinase and aspartyl proteinase, carboxypeptidases A and B, and leucine aminopeptidase [42, 44]. Maggots ES contain also allantoin, sulfhydryl radicals, calcium, cysteine, glutathione, embryonic growth stimulating substance, growth stimulating factors for fibroblasts, and other agents (Table 3) [42, 43]. The effect of wound healing stimulation is attributed to allantoin and urea. Ammonia, ammonium bicarbonate, and calcium carbonate contained in ES change medium reaction from acidic into alkaline, inhibiting bacterial growth. Also, tissue irritation by moving maggots also speeds the process of wound healing up [44]. Favourable effect on DFU healing is also associated with MT influence on disturbed mechanisms of the inflammatory process. Monocyte activity change via the cyclic AMP-dependent mechanism causes inhibition of secretion of proinflammatory cytokines TNF*α* and IL12p40 and the macrophage migration inhibitory factor (MIF) and increased secretion of antiinflammatory IL10. The process leaves phagocytosis untouched [75]. Maggots remove necrotic tissue and do not digest bones, tendons, or viable tissues. They offer a precise, accurate, and delicate debridement [44, 75, 76]. It seems that the level of wound debridement is not achievable with surgical methods. Maggots also clean off microorganisms. Based on the bacteriological analysis of the larval alimentary tract, it was observed that consumed bacteria die in further parts of the larval alimentary tract [77]. Maggots efficiently eradicate *Staphylococcus aureus*, including MRSA strains, Streptococci, and *Pseudomonas aeruginosa*. They have no effect on *Escherichia coli*, *Enterococcus*, *Proteus* [44, 47, 78, 79].

 MT may be ineffective in case of highly discharging or dried wounds. Insignificant side effects of the therapy include minor bleeding, pain, excessively induced exudates, increased body temperature, flu-like symptoms, allergic reaction, and skin maceration [44, 76, 80, 81]. There are practically no contraindications for MT in DFU therapy. Patient anxiety may constitute a limitation. *Lucilia sericata* maggot cultures are kept on sterile media, with sterile air flow, ensuring aseptic conditions. 1–3 mm long maggots are used for dressings. They are provided in two forms: open, for direct application on wound, or closed in a biobag. The network of the biobag is permeable and permits the migration of maggot ES to the wound. A wound qualified for MT requires no special preparation; edges of the wound are covered with an ointment protecting against digestive enzymes. Applied maggots, approximately 5–10 for one centimetre squared of the wound, may be covered by a nylon net, ensuring they remain in the wound area, and a humid dressing. A dry dressing is applied on top. As the outer dressing gradually becomes soaked, it should be changed [76, 77]. The dressing with maggots is maintained for 3 days, on average. After that period, the maggots should be removed by washing out the wound by saline. The procedure is repeated 1–4 times, if necessary. Closed dressings are more comfortable to use, because maggots cannot get out; however they often cannot be applied on deep DFU with small, irregular area [44, 45, 76, 82].

 The beneficial effects of using larvae in wounds were first noticed by Ambrose Paré in 1557. While treating battle wounds in Napoleon's army, Baron Larrey observed that maggots enhanced granulation formation. The first clinical application of maggot therapy was performed by J. F. Zacharias and J. Jones during the American Civil War [48].

 In 1931, Baer published results of his attempts to apply MT in therapy of osteitis in children [85]. However, sterility of maggots, their transport survival, and application of appropriate dressings constituted a problem. MT was difficult from the logistic point of view, costly, and burdened with a risk of infection, for example, with tetanus.

 Only in the 1980s, when the number of patients with chronic ulceration grew rapidly and standard therapeutic methods often proved inefficient, increased MT application was observed. Methods of sterile and industrial culture of maggots were developed, along with efficient transport and new types of dressings, which led to the application of MT in inpatient and outpatient settings.

 Since the beginning of the 21st century MT, called also the maggot debridement therapy (MDT), biosurgical debridement (BD) with maggots “biosurgeons,” or simply larval therapy, has been in the renaissance [76, 84].

 MT uses maggots fed on necrotic tissue only. They are *Lucilia sericata*, *Lucilia cuprina*, *Phormia regina,* and *Musca domestica*. The most commonly used species in MT is *Lucilia sericata. Lucilla sericata* maggots are highly voracious and mobile; they have a herd instinct and become even more active if they sense competition. In a natural environment, those flies lay their eggs in carrion. Maggots hatch on the next day. They raven for 5–7 days, grow, and pupate into mature flies in 10–14 days [76, 86].

DFU patients are a major indication for MT, and some comparative studies have been published. Jarczyk et al. [44] used MT in 4 DFU patients at risk of amputation, with nonhealing ulcerations (2–9 months). Complete healing of ulcers was achieved in 3 of those 4 patients. Sherman [45] compared the efficacy of conventional treatment (frequent changes of dressings, local antiseptics and antibiotics, hydrogel and hydrocolloid dressings, and surgical wound debridement) with larval therapy in patients with diabetic feet. After 5 weeks of therapy, wounds subjected to conventional treatment remained covered with necrotic tissue (33% of the surface of the wound), while in the case of larval therapy all the ulcerations were cleaned after 4 weeks. Another controlled cohort study demonstrated that MT was more effective and efficient in debriding DFU than the conventional therapy. MT was also associated with better wound granulation and epithelialisation [45]. Armstrong et al. [46] mentioned the benefits connected with larval therapy in the case of patients with peripheral vascular lesions and diabetic foot ulcerations. The previously mentioned authors investigated 60 patients, demonstrating more rapid wound healing, a threefold lower percentage of limb amputations (10% *versus* 33%), and shorter antibiotic therapy in patients subjected to biosurgical therapy, in comparison to the control group. Many other studies proved that MT may reduce time of therapy, reduce short-term morbidity, and reduce the risk of major amputation by as much as 50% in patients with DFU [44, 87–89].

Despite lack of unanimous evidence, clinical experience suggests that MT is effective and safe. Even more, MT used in wound bed preparation can be effective both clinically and financially, if used appropriately [90, 91].

Although indications for wound debridement with maggots are all chronic wounds and some acute ones, that logistically difficult therapy is best suited to DM patients in whom surgical excision of necrotic tissue, application of enzymatic preparations, and dressings speeding autolysis of necrotic tissue up is impossible, or if those methods are not sufficiently effective. In those conditions, MT seems to be a therapy of choice.

## 4. Autologous Platelet-Rich Plasma Therapy (PRPT)

 Making effective wound healing possible is the basic stage of the DFU curing process. Use of autologous platelet-rich plasma (PRP) in the form of local application of a gel obtained by centrifugation of full blood and addition of an activator, clotting agent, is designed for the creation of local conditions favourable to healing processes. PRP is defined as plasma fraction of autologous blood with a platelet count concentrated above the baseline [92]. It is a repository of growth factors, cytokines, adhesion molecules and clotting agents, and leukocytes.

Platelets contain numerous natural growth factors released from their *α* granulations and stimulating healing processes (Table 3). In 1974, Ross et al. [50] in the *in vitro* study noted thrombin-activated platelets as a source of growth factors that could initiate the body's natural healing. Added to platelet-poor plasma, they increased activity of smooth muscle cells and fibroblasts.

PRP is obtained by repeated centrifugation of autologous full blood [93]. The resulting concentrate, combined with activating bovine thrombin, forms a gel that seals the wound. The gel is placed on wound bed and protected by a cover dressing. The dressing may stay in place for up to 7 days.

 Various production systems are suggested, with various abilities of aggregation of platelets and leukocytes. For that reason, there are various preparations available, slightly different from each other [83, 94–96]. Sufficient cellular response to platelet concentrations first began when a 4-5-fold increase over baseline platelets' number was achieve, at least one million platelets per microlitre [97]. Platelets release over 30 factors responsible for healing processes, including three isomers of the platelet-derived growth factor (PDGF *αα*, *ββ*, *αβ*), vascular endothelial growth factor (VEGF), transforming growth factor-*β*1 (TGF-*β*1), transforming growth factor-*β*2 (TGF-*β*2), epidermal growth factor (EGF), insulin-like growth factor-1 (IGF-1), and others. PRP contains also proteins responsible for cellular adhesion, fibrin, fibronectin, vitronectin, and also osteocalcin and osteonectin [83, 98, 99]. PRP contains additionally leukocytes that increase its antibacterial properties and synthesize interleukins as part of a nonspecific immune response [95, 96]. PDGF is a growth factor found also in macrophages and endothelial cells. TGF is also found in macrophages, and EGF is present in macrophages, monocytes, and keratinocytes. VEGF is found mostly in endothelium, and IGF-1 is predominantly produced in the liver. Besides activity of leukocytes, the antibacterial effect of PRP is a result of activity of PGDF, by activation of macrophages, and VEGF, by stimulation of macrophages and monocytes. Re-epithelialisation and fibroblast proliferation are mostly the effect of PDGF, TGF, EGF, and IGF-1, stimulating the deposition of extracellular matrix (ECM). PDGF and IGF-1 are responsible for stimulation of other growth factors and cytokines, and angiogenic effect is shown mostly by VEGF, EGF, and PDGF [100, 101]. All these functions have been demonstrated through specifically designed *in vitro* experiments [51–53].

Platelets attach to the connective tissue, and growth factors are released via degranulation of *α* granulation in just 10 minutes after initiation of blood clotting processes. The majority of them is released during the first hour and is bound to membranous receptors in surrounding cells, activating intracellular signalling pathways.

Following a rapid release of growth factors, platelets contained in PRP synthesise and secrete their additional quantities for subsequent 7 days. After that time, the healing function is taken over by macrophages. Experimental and clinical studies demonstrated the most profound accelerating effect on wound healing in the 3rd week after PRP application [55, 102].

In 1982, Knighton et al. [56] in their experimental *in vivo* animal study demonstrated that a thrombin-activated autologous platelet concentrate stimulated neoangiogenesis, collagen synthesis, epithelial cells proliferation, fibroblast proliferation, and fibrin decomposition products stimulated leukocyte activity. Result of those experiments was a clinical study published four years later on the effective use of local PRP application and PRP-contained growth factors on therapy of chronic wounds, including DFU [54]. The percutaneous delivery of platelet-rich plasma (PRP) Gandhi et al. [61] used in the diabetic BB Wistar femur fracture model. PRP delivery at the fracture site normalized the early (cellular proliferation and chondrogenesis) parameters while improving the late (mechanical strength) parameters of diabetic fracture healing.

A complete clinical evaluation of PRP is still pending. Preliminary results of basic studies and preclinical and clinical trials have not been confirmed in large controlled studies. Available analyses in case reports [62, 67], case series [59, 64], randomised controlled studies [57, 60], cohort studies [58], and metaanalyses [65, 66] are based on small samples. However, they indicate that PRPT applied for nonhealing DFU is a more effective method compared to local conventional therapy.

In the multicentre study by Villela and Santos, and de Leon et al. [63, 66] on a group of 200 patients with 285 wounds, PRPT could restart the healing process in the majority of cases. Rapid treatment response was observed in 275 of 285 wounds, and the size of the reply was high with reported statistically significant outcomes. Carter et al. [65] completed a metaanalysis on the use of PRPT on wound healing patients with DFU, which led to the conclusion that autologous PRP gel promises as an effective treatment for severe DFU. Dougherty [103] statistically analysed efficacy versus cost and quality of life of a patient with DFU treated with PRP compared to conventional or other alternative therapeutic methods. Cost analysis completed in the group of over 200 thousand patients involved quality of life, recurrence, necessary amputation, and mortality. The study demonstrated the highest cost effectiveness with the consideration of quality of life for patients treated with PRP. According to the analysts, PRPT is potentially the most attractive alternative for DFU, that may reduce the cost burden and health effects of nonhealing DFU.

DFU was demonstrated to be associated with reduced activity of many growth factors—hence the concept of their exogenous supply. DM patients demonstrate deficiency of biological stimulators. It is a result of their metabolic and ischaemic problems, and that deficiency inhibits reparative processes and facilitates or intensifies development of infection.

Other studies demonstrated synergistic cooperation of growth factors contained in PRP, and their optimal proportions influence processes of normal healing [104–107].

Therefore, PRP seem to be an effective—if not the most effective—and safe preparation used for therapy of DFU. PRP is an autologous product, therefore constitutes no risk of viral hepatitis or HIV infection. Observed abnormal reactions to clotting activators are very rare [106].

PRPT is a rich source of locally active (bioregulating) growth factors and cytokines that improve conditions of wound healing. Relatively simple and cheap production of PRP argues for continued interest in that adjunct method. It seems that specific cellular therapy constitutes an additional and valuable option in therapy of DFU resistant to the conventional therapy.

## 5. Conclusions

 Prevention of DFU is the main point to reduce the associated high morbidity and mortality rates among patients with DM. DFU management involves a multidisciplinary approach from prevention (health educators) to treatment (diabetologist, surgeon, vascular surgeon, orthopaedist, radiologist, shoemaker, and kinesiotherapist).

 Proper, effective treatment of DFU prolongs life and improves its quality. The gold standard for DFU includes debridement of the wound, infection cure, revascularization when needed, and other new therapies [49].

 Among the adjunct DFU treatment methods are HBOT, MT, and PRPT. The new research on advanced therapy in DFU included stem cells, growth factors, skin substitutes, and gene therapy as well. The preliminary results are often promising, but randomised controlled trials are needed. When considering the adjunct treatment (HBOT, MT, PRPT, and others), it is clear that more patients suffered from refractory wounds, which lead to more frequent hospitalizations from sepsis, gangrene, amputation, and death. The combination therapy should improve the rate of healing and prolong the time of complications (Tables 4 and 5).

The conclusion of the International Working Group of the Diabetic Foot (IWGDF) systematic review is that with the exception of HBOT and possibly negative pressure wound therapy, there is little published evidence to justify the use of other therapies [49]. Conclusions were as follows.

1. DFU treatment should be strictly applied according to the gold standard accepted by the International Working Group on the Diabetic Foot [108, 109].
2. The gold standard for DFU treatment includes debridement of the wound, management of any infection, revascularization procedures when indicated, and off-loading of the ulcer [108].
3. HBO can be applied as an adjunctive therapy for patients with severe soft tissue foot infections and osteomyelitis who have not responded to conventional treatment, though the available data are insufficient [32, 71, 72, 109, 110].
4. Recent reports suggest that MT is effective in the elimination of drug-resistant pathogens [78, 84].
5. PRPT is reserved as a second-line therapy similar to HBO, especially in the treatment of refractory wounds [63, 102].

## Figures and Tables

**Table 1:** Therapeutic protocols used in the intervention and control groups in included studies focusing on the use of HBOT in DFU [5, 8, 32].

First author and year of publication	Study group (no. of patients)	Control group (no. of patients)
Löndahl et al., 2010 [33]	*N* = 49	*N* = 45
	Evaluation of wether adjunctive treatment with HBOT compared with treatment with hyperbaric air (placebo) would have any therapeutic effect HBOT a treatment period at 2.5 ATA for 85 min daily (session duration 95 min), five days a week for 8 weeks (40 sessions)	
	Complete healing of the index ulcer was achieved in 25/48 (52%), 3 major amputations, 4 minor amputations	Complete healing of the index ulcer was achieved in 12/42 (29%), 1 major amputation, 4 minor amputations

Duzgan, 2008 [34]	*N* = 50	*N* = 50
	Standard therapy (ST) + HBOT HBOT 2-3 ATA for 90 min/2 sessions per day, followed by 1 session on the following day; 33% were healed without surgery treatment; 16% (8) required operative debridement, an amputation, or the use of a flap or skin graft; 8% (4) underwent distal amputation; 0 required proximal amputation	ST daily wound care, dressing changes, local debridement, and control infection 0% of patients were healed without surgery treatment; 100% (50) required either operative debridement, an amputation, or the use of a flap or skin graft; 48% (24) underwent distal amputation; 34% (17) required proximal amputation

Abidia et al., 2003 [35]	*N* = 9 (100% oxygen)	*N* = 9(control—air)
	HBOT 2.4 atmospheres absolute (ATA) for 90 min daily, 5 days per week, totaling 30 sessions	2.4 atmospheres absolute (ATA) for 90 min daily, 5 days per week, totaling 30 sessions
	Complete epithelialization was achieved in 5 out of 8 ulcers; the median decrease of the wound areas was 100%	Complete epithelialization was achieved in 1 of the 8 ulcers; the median decrease of the wound areas was 52%

Kalani et al., 2002 [36]	*N* = 17	*N* = 21
	40–60 session of HBOT	Conventional treatment
	Investigation the long-term effect of HBOT, 76% of (13) patients had healed; 12% (2) were amputated	48% of (10) patients had healed, 33% (7) were amputated

Faglia et al., 1998 [19]	*N* = 51	*N* = 64
	Comparison therapy plus treatment in a multiplace HBO chamber Two phases: (1) first (antibacterial) phase uses 100% oxygen at 2.5 ATA for 90 minutes daily; (2) second (reparative) phase uses 100% oxygen at 2.2–2.4 ATA for 90 minutes, 5 days a week	Debridement, topical antimicrobial agents, and occlusive dressing. Empirical antibiotic therapy modified following sensitivity results. Diabetic control with insulin. PTCA or CABG, if needed

Zamboni et al., 1997 [18]	*N* = 5	*N* = 5
	Comparison therapy plus treatment in a monoplace HBO chamber with 100% oxygen at 2 ATA for 120 minutes, 30 sessions 5 days a week	Debridement, silver sulfadiazine dressing twice a day for 5 days, and culture-specific antibiotics

Faglia et al., 1996 [20]	*N* = 35	*N* = 33
	Comparison therapy plus treatment in a multiplace HBO chamber. Two phases: (1) first (antibacterial) phase uses 100% oxygen at 2.5 ATA for 90 minutes daily; (2) second (reparative) phase uses 100% oxygen at 2.2–2.4 ATA for 90 minutes, 5 days a week. Mean (SD) number of sessions = 38 (8)	Debridement, topical antimicrobial agents, occlusive dressing. Empirical antibiotic therapy modified following sensitivity results. Diabetic control with insulin. PTCA or CABG, if needed

Doctor et al., 1992 [37]	*N* = 15	*N* = 15
	Conventional management and 4 sessions of hyperbaric oxygen therapy HBO chamber with 100% oxygen at 3 ATA for 45 minutes, 4 sittings over 2 weeks	Regular surgical treatment, incision and drainage, debridement, local dressing with boric acid and bleaching powdered solution, or glycerine acriflavine Amputation for gangrene or infection above the knee Cephalosporins, aminoglycosides, and metronidazole with changes made following sensitivity patterns Diabetic control with insulin

Baroni et al., 1987 [17]	*N* = 18	*N* = 10
	Comparison therapy plus treatment in a multiplace HBO chamber. Two phases: (1) first (antibacterial) phase uses 100% oxygen at 2.8 ATA for 90 minutes daily; (2) second (reparative) phase uses 100% oxygen at 2.5 ATA for 90 minutes. Mean (SD) number of sessions = 34 (21.8)	Debridement. Diabetic control with insulin

**Table 2:** The Wagner classification of diabetic foot ulceration [15].

Grade	Clinical description
0	No open ulcer, high risk
1	Superficial ulcer with subcutaneous involvement
2	Deep ulcer with tendon or joint involvement
3	Deep ulcer with bone involvement
4	Wet or dry gangrene (forefoot), without cellulitis
5	Generalized (whole foot) gangrene

**Table 3:** Statement of research on MT and PRPT.

Methods	Cell cultures	Animal trials	Clinical trials
Maggot therapy (MT)	Prete, 1997 [42] Gupta, 2008 [43]		Jarczyk et al., 2008 [44] Sherman, 2003 [45] Armstrong et al., 2005 [46] Bowling et al., 2007 [47] Chan et al., 2007 [48] Game et al., 2012 [49]

Platelet-rich plasma therapy (PRPT)	Ross et al., 1974 [50] Cenni et al., 2005 [51] Kark et al., 2006 [52] Borzini and Mazzucco, 2007 [53]	Knighton et al., 1986 [54] Pietramaggiori et al., 2008 [55] Borzini and Mazzucco, 2007 [53]	Knighton et al., 1982 [56] Krupski et al., 1991 [57] Margolis et al., 2001 [58] McAleer et al., 2006 [59] Driver et al., 2006 [60] Gandhi et al., 2006 [61] Borzini and Mazzucco, 2007 [53] Scimeca et al., 2010 [62] Villela and Santos, 2010 [63] Frykberg et al., 2010 [64] Carter et al., 2011 [65] de Leon et al., 2011 [66] Slesaczeck et al., 2012 [67] Game et al., 2012 [49]

**Table 4:** Wound healing process and the alternative methods.

Elements of wound healing		Methods	
	HBOT	MT	PRPT
Inflammation	Bactericidal and bacteriostatic effects on both aerobic and anaerobic bacteria through the action of the super oxide enzyme*	Antibacterial potential effect of alkaline pH of maggot secretion [77, 78] Wound bacteria are killed as they pass through the maggot's digestive tract* Presence of a potent bactericide present in maggot secretions* Cytokine regulation and enhanced phagocytosis [75]	Suppresses cytokine release and limits the amount of inflammation, interacting with macrophages to improve tissue healing Enhances phagocytosis and chemotaxis [54]* Antimicrobial host defence enriched with growth factors and other active substances [83]*

Granulated tissue formation—epithelialization	Increases epidermal cells and fibroblast proliferation and differentiation [29]	The healing of wounds is an interactive process (regulators as growth factors, cytokines and chemokines) [42] Synthesized and released locally proteins or polypeptides [42, 43] Increases fibroblast proliferation through maggots excretions and secretions [75]	Influences on chemotaxis, mitogenesis, and differentiation Promotes healing by stimulating fibroblast and keratinocyte proliferation Promotes granulation tissue formation [55, 61]* and epithelialisation

Matrix formations	Increases fibroblast proliferation and collagen production	Stimulates extracellular matrix and remodeling processes [45]	Stimulates the deposition of extracellular matrix and collagen [56]*

Angiogenesis	The oxygen gradient promotes the formation of new vessels required for wound healing [28, 35, 68]	Growth factors, cytokines, and chemokines provide significant vasodilation and increased capillary permeability to the wound site, allowing the infusion of recruited polymorphonuclear leucocytes (PMNs) and macrophages [48, 84]	Promotes new capillary growth [55, 56]*

*Animal models.

**Table 5:** Clinical relevance in DFU according to the alternative methods.

Clinical relevance	Methods		
	HBOT	MT	PRPT
Reduced area in diabetic foot	Yes**	No	Yes**
Antiedema effect	Yes**	No	No
Decreased risk amputation	Yes**	Yes**	Yes
Shortening time of therapy	Yes**	Yes**	Yes**

**Clinical studies.
